# Mother-Child Play: A Comparison of Autism Spectrum Disorder, Down Syndrome, and Typical Development

**DOI:** 10.3389/fpsyg.2016.01829

**Published:** 2016-11-22

**Authors:** Arianna Bentenuto, Simona De Falco, Paola Venuti

**Affiliations:** Department of Psychology and Cognitive Science, University of TrentoRovereto, Italy

**Keywords:** Down Syndrome, Autism Spectrum Disorder, play skills, mother–child interaction

## Abstract

The purpose of the present study was to analyze mother-child collaborative play in children with Autism Spectrum Disorders (ASD) compared to children with Down Syndrome (DS) and typical developing children (TD). Children with ASD are often described as having deficient play skills, particularly in the symbolic domain. Caregivers’ involvement in child play activities increases the structural complexity of playing in both typically developing children and children with disabilities. Participants included 75 mothers and their children with ASD (*n* = 25), with down syndrome (*n* = 25) and with typical development (*n* = 25). Mother–child play sessions were analyzed using a coding system for exploratory and symbolic play. Results indicated that children with ASD showed more exploratory play compared to children in the other groups. No significant differences emerged between the three groups for child symbolic play or for mother play. These findings are discussed in relation to the debate about functional and symbolic play in children with ASD and in relation to the importance of setting and age for play assessment.

## Introduction

### The Development of Play

Play allows children to learn and practice new skills in supportive conditions and is essential for child development (Boucher, 1999). While engaging in play activities, children develop not only motor skills but also cognitive and social skills (e.g., Bornstein and O’Reilly, 1993; Venuti et al., 2008). Moreover, appropriate play behaviors provide the opportunity for social interaction and vice versa (Piaget, 1962; Bretherton, 1984; Hobson et al., 2009). Play shows a universal developmental path; from manipulative and functional exploration to symbolic and pretend play. Children at first typically involve in exploratory play activities which are tied more closely to the physical properties of toys, and later they engage in symbolic play actions which rely on representational abilities. More specifically, very young children’s object play is characterized by exploratory sensorimotor manipulation whose aim is to extract information about objects characteristics. Successively, children’s play shows symbolic qualities and begins to represent experiences.

The manifestation of symbolic representation in children’s play has been observed to occur between 12 and 24 months of age (Cote and Bornstein, 2009; Lillard et al., 2012). Symbolic play repertoires include simple pretense about self and about others, sequences of pretense, and substitutions in pretense. Pretend play is when a child projects a mental representation onto reality in a spirit of fun. When children pretend, three different cognitive skills can be observed: attributing proprieties to objects, making references to absent objects or places, using objects as something else (i.e., object substitution). The developmental trajectory of play is associated with the emergence of children new cognitive skills. Indeed, child play and developmental age tend to be strongly associated in typically and atypically developing children (Hill and McCune-Nicolich, 1981; Beeghly and Cicchetti, 1987; Tamis-Lemonda et al., 2002). Changes in play skills and cognitive development often co-occur, especially regarding the transition from concrete to symbolic functioning (El’Konin, 1999); language and symbolic play emerge concurrently, and important progresses in either domains emerge at similar points in the first 2 years of life (Spencer, 1996). Gould (1986) found that scores on the Bayley Scales of Infant Development were positively correlated with ratings of play and language abilities, concluding that while often overlapping greatly with non-verbal cognitive abilities, language represents a separate cognitive domain, and may have a unique role in the development of play. Several other studies have identified strong positive correlations between either receptive or expressive language and play in typical children and atypically developing children (e.g., Mundy et al., 1987; Lewis and Boucher, 1988; Spencer, 1996; Musatti et al., 1998).

Starting from the study of the developmental progression of play, Bornstein and O’Reilly (1993) operationalized a scale for play behavior that followed a progression from simple manipulation of toys, to recognition of conceptual relationships between objects (i.e., functional play and combinatory play), to increasingly decontextualized play (i.e., symbolic or pretend play). The results suggested that children’s play was a valid and reliable way to evaluate progressively complex and cognitively demanding behaviors (Lifter et al., 1993; Lifter, 2000).

### Play in Children with ASD

To date the specific characteristics of play behaviors of children with Autism Spectrum Disorder (ASD) have not been conclusively explored and are still at the center of a vivid scientific debate encompassing different and crucial topics such as diagnosis and intervention (Sigman and Ungerer, 1984; Williams et al., 2001; Hobson et al., 2009). Deviations in play behavior in children with ASD can be detected in the 1st year of life (Ungerer and Sigman, 1981; Van Berckelaer-Onnes, 2003) and are evident through all phases of play development. Pretend play deficits are so commonly recognized in ASD that a failure to use toys symbolically is considered in the main diagnostic systems for ASD (e.g., Autism Diagnostic Interview-Revised; Rutter et al., 2003) (Ungerer and Sigman, 1981; Sigman and Ungerer, 1984). Ungerer and Sigman (1981), Sigman and Ungerer (1984) reported that children with ASD had lower occurrence and less variety of functional play acts, less pretend doll play and shorter play sequences; therefore, indicating a limited capacity of symbolic play in children with ASD. Williams et al. (2001) found that when children with ASD show symbolic play they miss the spontaneous and innovative components of symbolic play which is instead replayed in a learned routine.

However, scientific studies on ASD have demonstrated difficulties at various levels of play and not only in pretense. Some studies have described the first phase of play development in children with ASD as characterized by a number of unusual features. Play behaviors of children with ASD are often focused on a limited selection of objects (Van Berckelaer-Onnes, 2003), or even on a specific part of a single object (Freeman et al., 1979). Some researches have shown that children with ASD produce the same number of functional acts as typically developing (TD) children under spontaneous as well as structured conditions (e.g., Baron-Cohen, 1987; Lewis and Boucher, 1988; Van Berckelaer-Onner, 1994; Charman and Baron-Cohen, 1997; Libby et al., 1998; Williams et al., 2001) but they spend significantly less time playing functionally than TD children (Sigman and Ungerer, 1984; Lewis and Boucher, 1988; Jarrold et al., 1996). Williams et al. (2001) found that children with ASD engaged in functional play acts on a small variety of objects compared to children with DS and TD children.

On the other hand, some studies found more similarities than differences in play of children with ASD when compared to typically and atypically developing peers. Dominguez et al. (2006) reported no differences in the proportions of functional or symbolic play in a group of children with ASD, related to TD children, matched on chronological age, however, children with ASD showed less interest in specific types of objects and more exploratory and sensorimotor play. Warreyn et al. (2005) examined spontaneous symbolic play in 3–6 years old children with ASD in interaction with their mothers compared to a control group (including children with language delay and children with a developmental delay) matched on age and IQ. They found similarities in the symbolic play level of children with ASD and mental-age matched children.

Some studies have found a relationship between language and symbolic play in children with ASD. For example, children with ASD who engaged in symbolic play had significantly higher verbal mental age than those who did not (Baron-Cohen, 1987). Sigman and Ungerer (1984) reported that receptive and expressive language were related to the play of children with ASD though only receptive language was related to play skills in children with typical development and children with intellectual disabilities.

### Maternal Behaviors during Play

The mother may play an important role on the quantity and quality of child play behavior (Naber et al., 2008). Children can improve their play skills with more competent partners, such as parents, who can imitate and prompt their children’s actions (Riquet et al., 1981; Sigman and Ungerer, 1984; Ginsburg, 2007).

Differences in children’s maturing cognitions and behaviors are mediated by their parents’ promotion of play and there is strong evidence that an adult partner’s participation in child play enhances its complexity, duration, and frequency (Bornstein et al., 1996, 2002; Venuti et al., 2008). Children spontaneously initiate play sequences, but they also imitate and learn from the play they see (e.g., Užgiris et al., 1989). Indeed, adults engage in many different roles in shaping children’s object and representational play; they themselves can play in ways children observe and learn from, they are able to induce play, and they can provide supports for play. Moreover, in demonstrating play, the mother provides her child with information about how to engage in particular activities by modeling the action. The mother can also use language to solicit the play of her child. In soliciting, a mother places the onus for play on the child by verbally encouraging (but not modeling) the child’s participation in specific play activities (Bornstein et al., 2002).

In spite of the limited social skills of children with ASD, mothers of children with ASD exhibit an equal number of social approaches to their child, and have been shown to be as sensitive and responsive as mothers of other intellectually delayed and TD children (Brooks-Gunn and Lewis, 1984; Doussard-Roosevelt et al., 2003; van Ijzendoorn et al., 2007). A relevant aspect emerging consistently in the literature on parental interaction with children with ADS is the directive style of the mothers. In line with this aspect, Kasari et al. (1988) examined parent–child interactions in children with ASD compared with children with other intellectual disabilities and developmentally matched typically developing children. The results showed that caregivers of ASD children were similar to the other caregivers in their responsiveness to child non-verbal communication bids and did not differ in their engagement in mutually sustained play. However, parents of children with ASD used control strategies more frequently than parents of TD children, and they held their children physically on task more often while mothers of children with intellectual disability pointed more often to objects. In this study (Kasari et al., 1988) individual differences within the ASD sample were found indicating that mothers regulated their children’s behavior less and showed more mutual play and positive feedback to more communicatively able children with ASD. In other studies, Lemanek et al. (1993), Doussard-Roosevelt et al. (2003), observing mothers and their preschool children with ASD in play sessions, reported that the quantity of parental initiatives did not differ from what was observed in mothers of typically developing preschoolers. However, mothers of children with ASD used more physical contacts, more high intensity behaviors, and fewer social verbal approaches with their children with ASD.

The main purpose of the present study was to analyze mother-child collaborative play in children with ASD. We aimed to look at several features of exploratory and symbolic play in children with ASD compared to a group of mental age-matched typically developing children (TD) and children with Down Syndrome (DS). We had the following aims and hypotheses:

(1) We aimed to analyze child exploratory play according to the different levels of its complexity. We expected that children with ASD would engage more in exploratory play compared to the control groups and especially in unitary functional activity, in line with their tendency to engage in repetitive behaviors.
(2) We aimed to analyze child symbolic play according to the different levels of its complexity. We expected that children with ASD would show less complex symbolic play behaviors compared to the control groups.
(3) We aimed to compare maternal play in the three groups during play in terms of mothers’ demonstrations and solicitations of the play. As mothers usually adapt their play to their children’s level of play, we expected that mothers of each group of children would follow the play of their children. We expected mother of children with atypical developmental (ASD or DS) to show more solicitations than mothers of children with TD because mothers of children with atypical developmental are often reported to be more directive.
(4) We aimed to analyze the associations between maternal demonstrations and solicitations and child play. Considering the well documented scaffolding role of mothers during collaborative play, we expected positive correlations between maternal and children behaviors.

## Materials and Methods

### Participants

A total of 75 children and their mothers participated in this study. The index group consisted of 25 children with ASD and their mothers. The control groups consisted of: (a) 25 mental-age-matched typically developing children and their mothers; (b) 25 children with DS and their mothers (See sociodemographic information in **Table 1**). All children with DS had the Trisomy 21 type, confirmed by chromosomal analysis. No mental age data were available for the control group, but interviews with parents, examination of health records, and observations during the study all indicated that they were developing typically. We also had two other converging kinds of data on children in the TD sample: (a) data on the Vineland Assessment of Behavioral Adaptation (Sparrow et al., 1984) showed that children fell within the normal range (*M* = 102.72, *SD* = 11.66), and (b) data from a longitudinal study showed that children had IQs in the normal range (*M* = 103.48, *SD* = 7.07) at 48 months. Multivariate analyses were conducted on chronological age, mental age and mother age. Only chronological age was significantly different between the three groups.

**Table 1:** Mean (M), standard deviation (SD), and coefficient of variation (CV) of the demographic characteristics.

Child and mother characteristics	Autism Spectrum Disorder			Down Syndrome			Typical development		
	*M*	*SD*	VC	*M*	*SD*	VC	*M*	*SD*	VC
Mental age (months)	24.21	9.82	0.38	21.12	4.39	0.22	20.01	0.21	0.01
Chronological age (months)	43.33	7.62	0.18	36.68	8.71	0.24	20.01	0.21	0.01
Mother’s age (years)	36.81	3.73	0.10	35.43	6.18	0.17	25.43	6.12	0.24
Social economic status	37.80	13.26	0.35	20.84	10.04	0.48	21.48	5.59	0.26

The diagnosis of participants with ASD was confirmed through clinical judgment by an independent clinician based on the DSM-IV-TR criteria (American Psychiatric and Association, 2000) for ASDs as well as through the Autism Diagnostic Observation Schedule (ADOS – Lord et al., 2002). Modules 1 and 2 were used for all the subjects and all the children passed the cut-off for the ASD. The Griffith Mental Developmental Scale (2nd Ed., Griffiths, 1996) was used to determine the developmental ages of children with atypical development. The socio-economic status (SES) of the families, calculated with the Four-Factor Index of Social Status (Hollingshead, 1975), indicated a low status in family with children with TD and family with children with DS; family with children with ASD showed a middle-low status. A group effect emerged between groups in socio-economical level *F*(2,72) = 4.2 *p* < 0.001. Informed consent was obtained from all parents. The study was approved by the Ethic Committee for Experiments Involving Human Beings of the University of Trento.

### Procedure

The present study followed a standardized protocol. Data were collected during 10-min play sessions video recorded continuously by a female observer. Observations took place at the Intervention Center in a small, quiet room, which was familiar to the participants. The findings of previous studies using 10-min observations of play lend credence to the validity of these temporal parameters (see Bornstein et al., 1996; Bornstein and Tamis-LeMonda, 1997; de Falco et al., 2008, 2010; Bentenuto, 2012).

During the session, the mother was asked to play with her child as she typically would with a set of standard, age-appropriate toys (doll, blanket, tea set, toy telephone, toy train, two small picture books, foam ball, and set of nesting barrels). This set of toys, used in previous researches (Bornstein et al., 2002; Tamis-Lemonda et al., 2002; de Falco et al., 2010) allows for different play behaviors ranging from exploration to pretense (see Bornstein and O’Reilly, 1993).

#### Play Code

As described in **Table 2**, the play code consisted of a mutually exclusive and exhaustive category system that included eight levels and a default (no play) category (see Bornstein and O’Reilly, 1993; Tamis-LeMonda and Bornstein, 1996); these play levels derived from previous researches on the progressive nature of play across the 1st years of life. Play was coded continuously by noting play levels as well as beginning times and end times (accurate to 1 s). Levels 1–4 constitute the macrocategory Exploratory play and Levels 5–8 constitute the macrocategory Symbolic play. For each level, four measures were calculated: the absolute frequency, the proportion frequency, the absolute duration, and the proportion duration. As these measures have been found to be consistently highly correlated in previous studies (see Bornstein et al., 1996), their mean standard score was used as a summary index representing the amount of each play level and each macrocategory (within each group of participants). The summary indexes, by considering frequencies and duration at the same time, controls the risk of results misinterpretation due to repetitive behaviors (high frequencies and short duration) or preservative behaviors (low frequency and long duration) known to occur in children with intellectual disabilities. Moreover, the use of the summary indexes, taking into account the proportion of exploratory/symbolic play of the total duration of the session, controls for differences in the time each child spent engaged in play during the observed sessions (range: 480–600 s). The play code was applied to the child’s play and also to the mother’s demonstrations and solicitations of play. In demonstrating play, the mother offers to her child information about how to engage in specific activities by modeling the action.

**Table 2:** Play coding scheme.

Play levels	Description
**Exploratory play**	
(1) Unitary functional activity	Production of effects to a single object (e.g., dialing a telephone)
(2) Inappropriate combinatorial activity	Inappropriate juxtaposition of two or more objects (e.g., putting the ball on the telephone)
(3) Appropriate combinatorial activity	Appropriate juxtaposition of two or more objects (e.g., putting the handset on the telephone base)
(4) Transitional play	Approximated pretense but without confirmatory evidence (e.g., putting the telephone handset to ear without vocalization)
**Symbolic play**	
(5) Self-directed pretense	Pretense activity directed toward self (drinking from an empty cup)
(6) Other-directed pretense	Pretense activity directed toward someone or something else (e.g., putting a doll to sleep)
(7) Sequential pretense	Linking two or more pretense actions (e.g., pouring into an empty cup from the teapot and then drinking)
(8) Substitution pretense	One or more object substitutions (e.g., pretending a cup is a telephone and talking into it)
**Default**	Not engaged in any of the above behaviors

#### Maternal Solicitations

In soliciting, the mother places the onus for play on the child by verbally encouraging (but not modeling) the child’s participation in specific play activities. Solicitations are defined as utterances which encourage the child to engage in a specific play activity when the child and mother are playing together (Bornstein et al., 2002). Each solicitation is coded for its level of play sophistication using the levels defined in the play code. Because solicitations are verbalizations, frequency measures (absolute and proportional) were calculated: the mean standard score (within each group of participants) of these two indices was used as a summary index representing the amount of either mother solicitations of exploratory or symbolic play. However, for the most part, solicitations only occur at play levels 1, 3, 5, and 6. Rarely would mothers solicit their children to perform inappropriate combinations (level 2), transitional play (level 4), sequences (level 7) or substitutions (level 8).

#### Interobserver Agreement

For each of the two codes, coding was carried out by two professional research assistants who were blind to hypotheses and purposes of the study and to additional information about the dyads (however, DS was easely detectable by visual inspection). Each coder was first trained to reliability (kappa; Cohen, 1960) on consensus coding. Average *kappas* between each pair of coders was calculated on 40% of the sessions and ranged from 0.74 to 0.81 for the Play code and from 0.75 to 0.82 for the Maternal Solicitations coding.

### Analytic Plan

We first conducted preliminary analyses of the data. Then, we reported descriptive statistics for child and for mother play in the three groups. To test our hypotheses about child play and mother play, one-way analyses of variance ANOVAs with group (ASD vs. DS vs. TD) as between-subject factor were used on the summary indexes of mother play and child play macrogategories. *Tukey post hoc tests* were used as *post hoc* tests and follow-up analyses on the eight play levels were carried out via separate ANOVAs, using Bonferroni *p*-value adjustment. Pearson correlation analyses were carried out to investigate associations between mother solicitation and child play.

## Results

### Preliminary Analyses

Prior to data analysis, all dependent variables and potential covariates were examined for normalcy, homogeneity of variance, outliers and correlations among variables (Fox, 1997). As noted in the description of the sample, group’s differences emerged in chronological age and social economic status so these variables were evaluated as potential covariate by examining their correlations with all dependent variables. No consistent patterns of significant correlations were found.

### Descriptive Statistics

**Table 3** presents descriptive statistics of play’s indexes for child play by group. **Table 4** present descriptive statistics for mother play by group. **Table 5** presents descriptive statistics for frequencies of maternal solicitation behaviors.

**Table 3:** Descriptive statistics for child play.

	Autism Spectrum Disorder *M* (*SD*)	Down Syndrome *M* (*SD*)	Typical development *M* (*SD*)
**Exploratory play**	0.29 (1.49)	-0.11 (0.66)	0.17 (0.83)
(1) Unitary functional activity	1.67 (2.09)	0.49 (0.78)	0.73 (0.86)
(2) Inappropriate combinatorial activity	-0.44 (0.30)	-0.38 (0.36)	-0.20 (0.62)
(3) Appropriate combinatorial activity	0.25 (1.3)	-0.15 (0.68)	0.36 (0.82)
(4) Transitional play	-0.31 (0.39)	-0.42 (0.28)	-0.19 (0.61)
**Symbolic play**	-0.18 (0.79)	-0.12 (0.68)	-0.05 (0.69)
(5) Self-directed pretense	0.07 (0.85)	0.02 (0.69)	0.23 (0.80)
(6) Other-directed pretense	-0.12 (0.92)	-0.29 (0.34)	-0.12 (0.42)
(7) Sequential pretense	-0.11 (0.74)	0.28 (0.96)	0.22 (0.86)
(8) Substitution pretense	-0.55 (0.00)	-0.52 (0.11)	-0.54 (0.09)

**Table 4:** Descriptive statistics for mother play.

	Autism Spectrum Disorder *M* (*SD*)	Down Syndrome *M* (*SD*)	Typical development *M* (*SD*)
**Exploratory play**	0.27 (1.32)	-0.08 (0.79)	0.22 (1.04)
(1) Unitary functional activity	1.58 (1.27)	0.69 (0.94)	0.82 (1.01)
(2) Inappropriate combinatorial activity	-0.56 (0.01)	-0.55 (0.06)	-0.33 (0.42)
(3) Appropriate combinatorial activity	0.33 (1.36)	0.09 (0.75)	0.99 (1.19)
(4) Transitional play	-0.48 (0.19)	-0.55 (0.06)	-0.44 (0.25)
**Symbolic play**	-0.12 (0.77)	-0.08 (0.76)	-0.22 (0.65)
(5) Self-directed pretense	-0.40 (0.32)	-0.31 (0.50)	-0.33 (0.50)
(6) Other-directed pretense	0.38 (1.14)	0.24 (0.93)	0.07 (0.66)
(7) Sequential pretense	0.05 (0.67)	0.26 (0.89)	-0.11 (0.89)
(8) Substitution pretense	-0.50 (0.23)	-0.46 (0.22)	-0.51 (0.19)

**Table 5:** Descriptive statistics for maternal solicitation behaviors.

	Autism Spectrum Disorder *M* (*SD*)	Down Syndrome *M* (*SD*)	Typical development *M* (*SD*)
**Exploratory play**			
(1) Unitary functional activity	3.68 (3.67)	5.88 (7.38)	4.24 (3.85)
(2) Inappropriate combinatorial activity	-	-	-
(3) Appropriate combinatorial activity	3.20 (4.91)	2.68 (4.85)	3.76 (6.52)
(4) Transitional play	-	-	-
**Symbolic play**			
(5) Self-directed pretense	4.76 (5.59)	4.68 (3.71)	8.68 (5.35)
(6) Other-directed pretense	5.96 (6.19)	7.24 (6.19)	4.28 (3.73)
(7) Sequential pretense	0.36 (0.76)	0.20 (0.50)	0.36 (0.86)
(8) Substitution pretense	-	-	-

#### Child Play

Results showed a statistical difference among children for exploratory play, *F*(2,72) = 3.9; *p* < 0.05). Tukey HSD *post hoc* indicated that children with ASD showed more exploratory play than TD children and children with DS (**Table 3**). No main effect of group emerged for symbolic play. Follow-up ANOVAs on the eight individual play levels did not yield any significant result after Bonferroni *p*-value adjustment. However, a tendency of children with ASD to display more Unitary Functional Activity than children in the control groups emerged; specifically, we found an effect of group on Unitary Functional Activity at a standard level of significance, *F*(2,72) = 3.3; *p* < 0.05), and Tukey HSD *post hoc* indicated that children with ASD performed more Unitary Functional Activity than both groups of control peers.

#### Maternal Play and Solicitation

Results showed a statistical difference among mothers for exploratory play, *F*(2,72) = 3; *p* < 0.05). Tukey HSD *post hoc* indicated that mothers of children with ASD showed more exploratory play than mothers of TD children and children with DS (**Table 4**). No significant differences between groups were found for maternal symbolic demonstration of the play.

ANOVAs yielded a significant group main effect for mother solicitation. Results showed that mothers of children with ASD used less symbolic solicitation than mothers of children with TD (**Table 5**). No significant differences between the three groups of mothers emerged in exploratory solicitation.

### Mother Solicitation/Demonstration and Child Play

Pearson correlation analyses showed strong positive associations between mother play and child play for all of the groups of children. More specifically, we found a high positive correlation between mother play and child play for exploratory play (TD: *r* = 0.43 *p* < 0.01; DS: *r* = 0.66 *p* < 0.01; ASD: *r* = 0.69 *p* < 0.01) and for symbolic play (TD: *r* = 0.37 *p* < 0.01; DS: *r* = 0.39 *p* < 0.01; ASD: *r* = 0.40 *p* < 0.01). Regarding the verbal solicitation a significant positive association emerged with exploratory index in normal developing children only (*r* = 0.21, *p* < 0.05) and with symbolic index in children with ASD only (*r* = 0.36 *p* < 0.01). No statistically significant associations were found between maternal solicitation and child play in children with DS.

## Discussion

Play is universally a crucial activity for the development of children. Through play, the child explores the physical characteristics of the objects and develops his/her cognitive skills (Bornstein and O’Reilly, 1993). Empirical studies of child objects and representational play have defined a normative trajectory of development: play activities of greater sophistication are gradually achieved in accordance with a normative developmental path that proceeds from exploration of objects to pretense with them (Belsky and Most, 1981; Tamis-LeMonda and Bornstein, 1996; Bornstein et al., 2002). To reach higher levels of sophistication, in addition to child developing cognitive abilities, the participation of a mature partner’ in play is fundamental. The purpose of the present study was to investigate several features of play in children with ASD compared to a group of mental age-matched TD children and children with DS during mother–child interaction. Specifically, we aimed to compare the three groups of dyads in terms of the structure of child play, quantity and quality of maternal play, and the associations between mother solicitation and child play.

Considering our first aim, we found that children with ASD were more engaged in exploratory activity compared to children with DS and children with TD. This result is in accordance with a previous study of Dominguez et al. (2006) showing that children with ASD engaged in more exploratory play than children with typical development. In particular, our results showed a tendency in children with ASD to spend more time in a “unitary functional activity,” that is the simplest exploratory play level, than the control groups. In other words, children with ASD engaged longer in simple activities including one object at the time, such as pushing the train or throwing a ball, compared to their peers. Unitary functional play activity requires a simple understanding of the objects’ properties and is less dependent from learning by an adult as a model; therefore, it can be suggested that this kind of play is easier for children with social impairments such as ASD (Thiemann-Bourque et al., 2011). On the other hand, this result can be explained with the tendency of children with ASD to engage with toys in repetitive ways, accordingly to the repetitive and stereotypical behaviors that characterize these disorders (Jarrold et al., 1993). Another possible explanation may rely on the sensory stimulation that these children get from some of these play activities (e.g., pushing the train); previous researches indicate that children with disabilities show preferences for toys that produce sensory feedback when used by a child. These preferences may be related to the ability of the toy to provide the child with structure through an external stimulus (Malone and Langone, 1994).

With respect to our second aim, we did not find any difference among the three groups of children concerning symbolic play. In our study children with ASD showed the same capacity for symbolic play of developmentally matched children with DS and TD children. This result is in contrast with traditional studies that showed abnormalities in symbolic play in children with ASD (Jarrold et al., 1993; Williams et al., 2001) and with the clinical evidence of affected symbolic play in children with ASD. As an example, Jarrold et al. (1996) in an experimental setting found that children with ASD aged 4–12 years produced less pretend play and, even when prompted, they produced pretend actions at a lower rate, compared to children with learning difficulties. The latter study differs from the current study for the older age range of the participants and for the structured setting. Mundy et al. (1986), who considered participants of about 24 months of mental age – similarly to or study – and tested free and structured play conditions, also reported abnormal symbolic play in children with ASD compared to mental age-matched children with intellectual disability, but the difference between groups was evident for structured symbolic acts only. On the other hand, our results accords with some more recent studies that have also indicated that children with ASD show the same pretend play abilities as typically developing children and children with other disorders matched on age and/or IQ (Warreyn et al., 2005; Dominguez et al., 2006). We believe that two main aspects contributed to the explanation of our findings: the developmental age of the children in our sample and the collaborative play situation we analyzed. First of all, the capacity for symbolization increases after 24 months of mental age; for this reason, the difficulties in symbolic play of children with ASD may become more evident at a developmental age of 2 years or more, whereas children in our sample had a developmental age of 21 months. Consistently, our code for the evaluation of symbolic play included also very simple acts of pretense directed to the self or to the others (drinking from a cup or make the mother drink from a cup) that could happen one at the time or chained into a sequence; object substitution, the highest level of symbolic activity had a very low frequency. In many other studies, instead, only imaginative and more spontaneous activity are considered. Also, qualitative indicators of fun and enjoyment were not accounted in our study. By the way, children with ASD in our study have a higher chronological age than typically developing children, therefore these children do present a limited capacity for symbolic play compared to their peers but we cannot ascribe it to a specific deficit that goes beyond the intellectual delay (Naber et al., 2008). The simplest levels of symbolic play seems to be achieved by children with ASD of our sample, consistently with studies demonstrating that symbolic and pretend play can develop in children with ASD at a slower pace, but for many of them pretend play stays qualitatively different from that of their typically developing peers, lacking qualitative indicators of fun and enjoyment (Riquet et al., 1981; Lewis and Boucher, 1995; Jarrold et al., 1996; Charman and Baron-Cohen, 1997; Rutherford et al., 2006; Kasari et al., 2013). This result can be also connected with some literature which found that when children with ASD receive prompts to perform, they engage in the same level of pretend play as typically developing children at the same developmental level (Lewis and Boucher, 1995; Charman and Baron-Cohen, 1997; Lewis et al., 2000). It can be hypothesized that in this early stage of symbolic play emergence, the presence of an attuned and supportive partner allowed the children the perform at the higher level of they capabilities within the Zone of Proximal Development (Vygotsky, 1978). Indeed, in our study, no associations of chronological and mental age with child play level emerged within the three samples of participants. Other factors, related to the interactive play setting we analyzed, might better account for the individual differences in children’s play, as described below.

Concerning the third aim, our expectations about mother play was confirmed: mothers adapted well their play activities to their child’s play sophistication level. In fact, mothers in all groups showed more exploratory play than symbolic play, as their children did. Moreover, just like their children, mothers of children with ASD presented more exploratory play but the same amount of symbolic play than mothers in the control groups. Thus, all mothers preferred to reinforce the kind of play behaviors in which their children showed better abilities, i.e., exploratory play. However, considering maternal stimulation through verbal solicitation, we found that mothers of children with ASD used fewer symbolic solicitations than mothers of children with TD, indicating that they didn’t encourage them to higher level of play, as mothers of typically developing children did. One possible explanation is that mothers of children with ASD already expected difficulties in the symbolic domain either for having experienced them at a different level or because they know they are diagnostic features of ASD. In contrast to the previous studies (Lemanek et al., 1993; Doussard-Roosevelt et al., 2003) our results did not show a greater verbal solicitation of mothers of children with ASD or DS.

Regarding the associations between maternal solicitation and child play, we found high correlations between mother and child play in all groups. These similarities, again, speaks for the ability of all mothers to appropriately adapt their play activity to child developmental level, independent from the child diagnostic condition (Kasari et al., 1988). Moreover, we found a strong positive correlation between maternal verbal solicitation of symbolic play and children actual symbolic play for mothers of children with ASD only. This result suggests that, like all mothers, mothers of children with ASD play a crucial role in their psychological development, but they might contribute even more to determine their actual achievements reducing the effect of the potential limitations imposed by their pervasive deficits (Flippin and Watson, 2011).

Future research is needed to examine more deeply the types of behaviors mothers use to match or scaffold their child’s play. Several limitations of this study should be mentioned. First, having larger samples would allow a greater generalization of the data. Second in our study we used an observation at single point, so longitudinal studies are needed to follow the development of these children’s play across time. Future work should also consider associations between parents’ positive affect and sensitivity and the level of specific abilities child’s play (Bornstein et al., 2002; Venuti et al., 2008; de Falco et al., 2010). However, this study has some important clinical implications. First of all, in a natural interactive setting, symbolic play of children with ASD was similar to that of developmentally matched children with DS and of TD children. It appears that simple levels of symbolic play skills are preserved in children with ASD with a developmental age of about 2 years and can be observed during a natural play interaction with a significant partner. Developmental age and context of testing condition appear therefore to be crucial variables for the comprehension of play skills development in children with ASD. Moreover, mothers of children with ASD were able to adapt to their children’s play level and to efficiently prompt appropriate play behaviors through language. These results speak in favor of early interventions for ASD that involve parent-child dyads and promote the development of social and cognitive skills within play interactions.

## Author Contributions

All authors listed, have made substantial, direct and intellectual contribution to the work, and approved it for publication.

## Conflict of Interest Statement

The authors declare that the research was conducted in the absence of any commercial or financial relationships that could be construed as a potential conflict of interest.
